# AGE DIFFERENCES IN STRESSOR REACTIVITY AND RECOVERY

**DOI:** 10.1093/geroni/igad104.0113

**Published:** 2023-12-21

**Authors:** Alyssa Minton, Christian Waugh, Jason Snyder, Susan Charles, Claudia Haase, Joseph Mikels

**Affiliations:** DePaul University, Chicago, Illinois, United States; Wake Forest University, Winston Salem, North Carolina, United States; DePaul University, Chicago, Illinois, United States; University of California, Irvine, Irvine, California, United States; Northwestern University, Chicago, Illinois, United States; DePaul University, Chicago, Illinois, United States

## Abstract

Strength and vulnerability integration theory (SAVI; Charles, 2010) posits that age differences in emotional experiences vary based on the distance from an emotionally eliciting event. Before and after a stressor, SAVI predicts that older age is related to motivational strivings that often result in higher levels of well-being. During stressor exposure, however, age effects are predicted to be attenuated or disappear completely. The present study examined how younger (n = 85; M age = 22.56 years) and older (n = 85; M age = 71.05 years) adults reacted to and recovered from a cognitive stressor using repeated positive and negative emotion probes. Results showed that both age groups were negatively impacted by the stressor, and both reported an initial boost in recovery afterward. However, older adults continued to recover across the recovery period compared to younger adults. This work elucidates that older adults are significantly impacted by stress but exhibit a resounding recovery.

